# Multiparametric Quantitative Ultrasound as a Potential Imaging Biomarker for Noninvasive Detection of Nonalcoholic Steatohepatitis: A Clinical Feasibility Study

**DOI:** 10.3390/diagnostics15172214

**Published:** 2025-09-01

**Authors:** Trina Chattopadhyay, Hsien-Jung Chan, Duy Chi Le, Chiao-Yin Wang, Dar-In Tai, Zhuhuang Zhou, Po-Hsiang Tsui

**Affiliations:** 1 Department of Medical Imaging and Radiological Sciences, College of Medicine, Chang Gung University, Taoyuan 333323, Taiwan; trinachatterjee93@gmail.com (T.C.); jsz82513@gmail.com (H.-J.C.); duychi346@gmail.com (D.C.L.); everybady123@gmail.com (C.-Y.W.); 2 Radiology Center, 103 Military Hospital, Vietnam Military Medical University, Hanoi 12108, Vietnam; 3 Department of Gastroenterology and Hepatology, Chang Gung Memorial Hospital at Linkou, Taoyuan 333423, Taiwan; tai48978@cgmh.org.tw; 4 Department of Biomedical Engineering, College of Chemistry and Life Science, Beijing University of Technology, Beijing 100124, China; 5 Division of Pediatric Gastroenterology, Department of Pediatrics, Chang Gung Memorial Hospital at Linkou, Taoyuan 333423, Taiwan

**Keywords:** quantitative ultrasound, nonalcoholic steatohepatitis, FibroScan, fatty liver disease

## Abstract

**Objectives**: The FibroScan–aspartate transaminase (AST) score (FAST score) is a hybrid biomarker combining ultrasound and blood test data for identifying nonalcoholic steatohepatitis (NASH). This study aimed to assess the feasibility of using quantitative ultrasound (QUS) biomarkers related to hepatic steatosis for NASH detection and to compare their diagnostic performance with the FAST score. **Methods**: A total of 137 participants, comprising 71 individuals with NASH and 66 with non-NASH (including 49 normal controls), underwent FibroScan and ultrasound exams. QUS imaging features (Nakagami parameter *m*, homodyned-K parameter *μ*, entropy *H*, and attenuation coefficient *α*) were extracted from backscattered radiofrequency data. A weighted QUS parameter based on *m*, *μ*, *H*, and *α* was derived via linear discriminant analysis. NASH was diagnosed based on liver biopsy findings using the nonalcoholic fatty liver disease activity score (NAS). Diagnostic performance was evaluated using the area under the receiver operating characteristic curve (AUROC) and compared with the FAST score using the DeLong test. Separation metrics, including the complement of overlap coefficient, Bhattacharyya distance, Kullback–Leibler divergence, and silhouette score, were used to assess inter-group separability. **Results**: All QUS parameters were significantly elevated in NASH patients (*p* < 0.05). AUROC values for individual QUS features ranged from 0.82 to 0.91, with the weighted QUS parameter achieving 0.91. The FAST score had the highest AUROC (0.96), though differences with the weighted QUS and homodyned-K parameters were not statistically significant (*p* > 0.05). Separation metrics ranked the FAST score highest, closely followed by the weighted QUS parameter. **Conclusions**: QUS biomarkers can be repurposed for NASH detection, with the weighted QUS parameter offering diagnostic accuracy comparable to the FAST score and serving as a promising, blood-free alternative.

## 1. Introduction

Nonalcoholic fatty liver disease (NAFLD), now the most common cause of chronic liver disease worldwide [1], encompasses a wide clinical spectrum ranging from simple steatosis to nonalcoholic steatohepatitis (NASH), which can further progress to fibrosis, cirrhosis, and hepatocellular carcinoma (HCC) [2]. NASH is histologically defined by hepatic steatosis, lobular inflammation, and hepatocellular ballooning [3]. Approximately 25% of individuals with NAFLD advance to NASH, and among those, up to 20% may progress to cirrhosis or HCC within two to three decades [4], underscoring the importance of early and precise diagnostic strategies.

While liver biopsy remains the gold standard for diagnosing NASH, its invasive nature, procedural risks, and susceptibility to sampling errors limit its utility in routine clinical settings. To mitigate these challenges, various noninvasive biomarkers have been proposed [5,6]. However, these biomarkers often exhibit inconsistent performance due to interindividual variability, including the presence of comorbidities, which hampers their effectiveness in confirming a NASH diagnosis.

Medical imaging provides a noninvasive and direct means of evaluating liver tissues. Among various modalities, ultrasound-based techniques are widely accessible and cost-effective. FibroScan, the most commonly used ultrasound method dedicated to the liver disease examinations, offers the controlled attenuation parameter (CAP) for steatosis assessment and liver stiffness measurement (LSM) for fibrosis evaluation [7]. The FibroScan–aspartate transaminase (AST) score (FAST score), which combines CAP, LSM, and AST, further allows NASH detection [8]. However, as it is derived from FibroScan parameters, the FAST score is still constrained by inherent limitations, such as fixed-depth single-point measurements and susceptibility to confounding factors like obesity [9], inflammation [10], and venous congestion [11].

Given that hepatic fat infiltration is a core pathological hallmark of NASH and a primary driver of disease progression, imaging biomarkers capable of quantifying steatosis have attracted increasing attention. Fat accumulation not only characterizes the early stages of NAFLD but also initiates metabolic and inflammatory processes that culminate in NASH. Therefore, techniques that can sensitively and accurately quantify hepatic steatosis may be instrumental in identifying NASH at an earlier and potentially reversible stage. Quantitative ultrasound (QUS) techniques, such as speed of sound [12], backscatter coefficient [12], attenuation [12,13,14], and envelope statistics imaging [15,16,17], have demonstrated promise in characterizing liver fat content with improved performances compared to conventional B-scan. Attenuation imaging measures the frequency-dependent loss of ultrasound energy, while envelope statistics describes the statistical distribution of backscattered echo amplitudes to capture tissue microstructural changes. Despite their growing use in steatosis grading, these techniques have not yet been widely applied or validated specifically for NASH diagnosis, leaving a significant opportunity for clinical advancement.

In this study, we aimed to validate the hypothesis that ultrasound imaging biomarkers of hepatic steatosis (QUS attenuation and envelope statistics imaging) can be effectively applied to the evaluation of NASH. The subsequent sections describe our experimental design, results, and discussion. Our findings demonstrate that QUS techniques can effectively identify biopsy-confirmed NASH, highlighting their potential as an alternative to the FAST score in the assessment of fatty liver disease.

## 2. Materials and Methods

### 2.1. Subject Enrollment

This study was approved by the hospital’s Institutional Review Board for the reuse of ultrasound data originally collected in a previous study [18] for academic research purposes. All participants provided written informed consent, and all procedures were conducted in accordance with approved ethical guidelines. An initial cohort of 97 patients was enrolled based on the inclusion criterion of a scheduled liver biopsy, either as part of a partial liver resection or for the clinical evaluation of chronic hepatitis. Patients were excluded if they were on current medication or had any coexisting acute or chronic illnesses.

All enrolled participants underwent anthropometric assessments, and fasting venous blood samples were collected to measure AST and alanine aminotransferase (ALT) levels. Liver biopsies were performed to confirm the presence or absence of NASH. Histological diagnosis was based on the NAFLD activity score (NAS), which incorporates steatosis (0–3), hepatocyte ballooning (0–2), and lobular inflammation (0–3) [19]. Patients with a NAS score of ≤3 were categorized as non-NASH, and those with a score ≥5 as NASH [20]. A total of 9 individuals with an indeterminate NAS of 4 were excluded, resulting in a biopsy-confirmed cohort of 88 patients, including 71 with NASH and only 17 with non-NASH.

**Figure 1:**
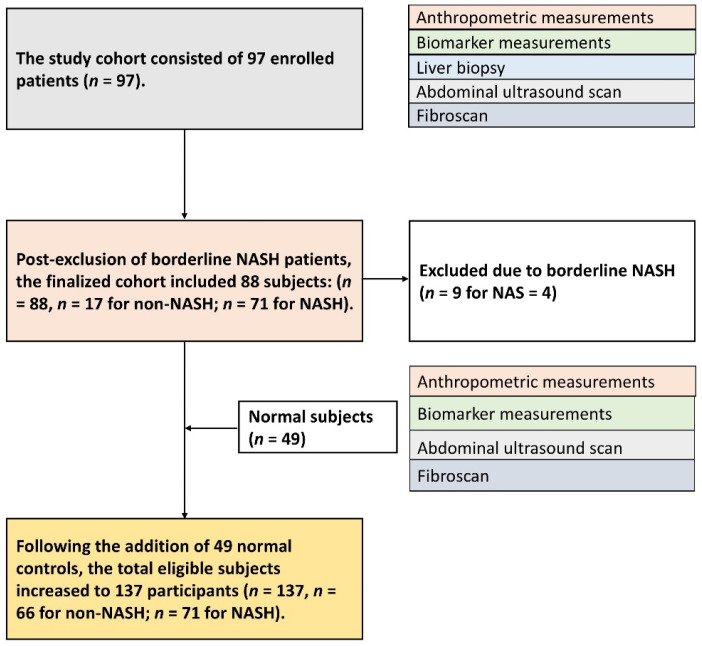
Subject enrollment process. The final study cohort consisted of 137 participants: 66 in the non-NASH group and 71 in the NASH group. NASH: nonalcoholic steatohepatitis; NAS: NAFLD activity score.

The number of biopsy-confirmed non-NASH cases was limited, which may have affected the statistical power of the analysis. To address this data imbalance, an additional 49 healthy individuals without known liver disease or chronic hepatitis were recruited as normal controls. These participants underwent the same anthropometric and laboratory assessments, and normal AST/ALT levels (<40 U/L) were used to confirm the absence of hepatic inflammation or injury. After including the normal control group within the non-NASH category, the final study cohort comprised 137 subjects: 66 in the non-NASH group and 71 in the NASH group. The subject enrollment process is illustrated in Figure 1.

### 2.2. FibroScan Measurement

Each subject underwent FibroScan assessment (Model 502 Touch, Echosens, Paris, France) while in the supine position. Measurements were obtained through the intercostal spaces using the M probe, targeting the right hepatic lobe. CAP and LSM values were recorded as the median of all valid measurements. Validity was defined as at least 10 successful acquisitions, a success rate greater than 60%, and an interquartile range (IQR)-to-median ratio below 30%. The FAST score was subsequently calculated using CAP, LSM, and AST values, following the regression formula established in a previous study [8].

### 2.3. Ultrasound Backscattered Data Acquisition

Abdominal imaging was performed using a clinical ultrasound scanner (Model 3000, Terason, Burlington, MA, USA) equipped with a convex array transducer (Model 5C2A, Terason Burlington, MA, USA). The transducer had a pulse length of approximately 2.3 mm and a central frequency of 3.5 MHz. Scanning parameters were standardized across subjects, with the focal length set to 4 cm, imaging depth to 8 cm, and imaging width to 12 cm. An experienced radiologist performed three scans per subject, carefully avoiding acoustic shadowing artifacts and large blood vessels to ensure high-quality data acquisition. Intercostal scanning was selected as the optimal approach for evaluating hepatic backscatter. At the moment of breath-hold during gentle, normal respiration, raw image data were acquired, each consisting of 128 backscattered radiofrequency (RF) signals sampled at 12 MHz.

### 2.4. Envelope Statistics and Attenuation Imaging

First, envelope images were generated by computing the absolute values of the Hilbert transform applied to each raw ultrasound dataset. The resulting ultrasound B-mode images were displayed as log-compressed envelope images with a dynamic range of 40 dB.

Envelope statistics images were then computed using a sliding window technique applied to the uncompressed envelope data [21]. Three parametric models were employed: the Nakagami parameter *m*, estimated via the moment estimator [22]; the scatterer clustering parameter *µ* from the homodyned-K (HK) distribution, estimated using the XU estimator [23]; and information entropy *H*, derived from the envelope histogram [24]. These models were selected based on their complementary theoretical characteristics and practical advantages in ultrasound tissue characterization. The HK distribution is a generalized model capable of accurately describing the statistical properties of ultrasound backscattered signals from soft tissues, accounting for both coherent and diffuse scattering signal components [24]. The Nakagami distribution, while mathematically simpler, serves as a computationally efficient approximation of the HK model and is widely used in clinical and experimental QUS studies [24]. In contrast, entropy provides a non-parametric, model-free approach, quantifying the statistical randomness of echo amplitudes without relying on any prior distributional assumptions [24]. This makes entropy particularly useful in heterogeneous tissue environments where parametric model assumptions may not hold.

In parallel, attenuation imaging was performed using the modified reference frequency method (RFM) applied to raw RF data with sliding window processing. In this study, we implemented an iterative reweighted least squares (IRLS)-based version of the RFM algorithm to estimate the attenuation coefficient *α* [25]. This approach enhances the precision of attenuation coefficient estimation by dynamically adjusting weight factors during the regression process, which mitigates the influence of noise and outliers in the RF signal. This feature is particularly advantageous for small-window attenuation imaging, where limited data within each window can increase estimation variability. By incorporating IRLS, the method achieves improved robustness and accuracy, enabling finer spatial resolution in attenuation mapping [26].

**Figure 2:**
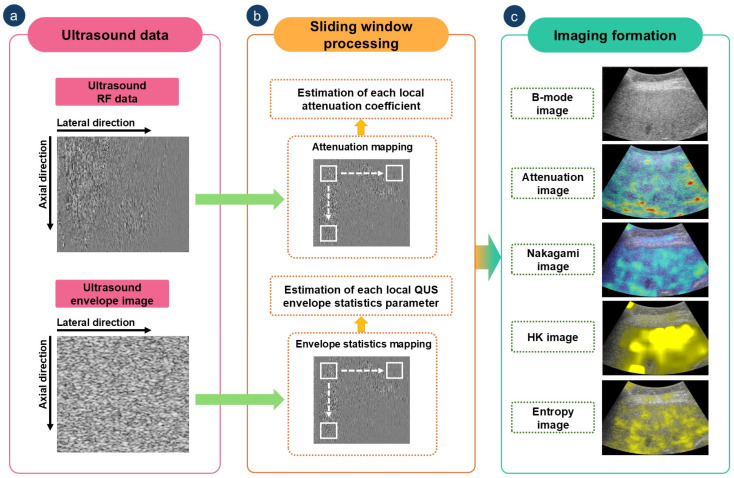
Algorithmic workflow for quantitative ultrasound (QUS) envelope statistics and attenuation imaging. QUS envelope statistics maps were generated using a sliding window technique applied to uncompressed envelope data, while attenuation images were constructed from raw radiofrequency (RF) signals using the modified reference frequency method. HK: homodyned-K. (**a**) Ultrasound data acquisition; (**b**) Sliding window processing; (**c**) QUS envelope statistics imaging.

A 50% overlapping ratio was used for all sliding window operations. Window sizes were set to three [22], five [23], and one [24] times the transducer pulse length for Nakagami, HK, and entropy imaging, respectively. For attenuation imaging, the window side length was set to nine wavelengths, allowing for sufficient spatial averaging to ensure robust attenuation coefficient estimation while preserving spatial resolution [25]. To combine anatomical and functional information, envelope statistics and attenuation maps were superimposed on B-mode images using pseudocolor encoding. The complete algorithmic workflow is illustrated in Figure 2.

### 2.5. Statistical Analysis

For each subject, a region of interest (ROI) was manually selected on the B-mode image of the liver parenchyma by a radiologist who was blinded to clinical information and had extensive experience in imaging anatomy. The same ROI was then applied to the QUS envelope statistics and attenuation images to extract the average values of the parameters *m*, *µ*, *H*, and attenuation coefficient *α*. ROI selection aimed to maximize coverage of the liver parenchyma while avoiding large vessels and non-hepatic structures, thereby minimizing estimation bias. To simultaneously evaluate the performance of multiparametric QUS, a weighted QUS parameter based on *m*, *µ*, *H*, and *α* was derived using linear discriminant analysis (LDA), which is used to identify a linear combination of input features that maximizes the separation between classes while minimizing within-class variance [26]. Box plots were used to present the median and IQR of each parameter for the non-NASH and NASH groups. Group differences were evaluated using an independent *t*-test. Diagnostic performance was assessed using receiver operating characteristic (ROC) curve analysis, and the area under the ROC curve (AUROC) was reported along with other relevant statistical metrics. Statistically significant differences in AUROC values were assessed using the DeLong test.

In addition to ROC and DeLong tests, we employed four complementary separation metrics for each parameter: (1) 1–overlap coefficient (OVC), indicating distributional separation; (2) Bhattacharyya distance, assessing distribution similarity; (3) Kullback–Leibler (KL) divergence, measuring information gain between groups; (4) silhouette score, reflecting intra-group compactness and inter-group separation. These metrics provided a comprehensive assessment of group separability from distributional and clustering perspectives. All analyses were conducted using Matlab software (Version R2022a, MathWorks, Natick, MA, USA), with statistical significance set at *p* < 0.05.

## 3. Results

Table 1 summarizes the demographic and clinical characteristics. Compared to non-NASH patients, the NASH group had significantly higher steatosis (2.61 ± 0.56 vs. 1.12 ± 0.48), ballooning (1.46 ± 0.58 vs. 0.47 ± 0.51), and inflammation scores (1.67 ± 0.50 vs. 0.88 ± 0.49; all *p* < 0.05). LSM, CAP, AST, and ALT values progressively increased from controls to non-NASH to NASH patients (all *p* < 0.05). Control subjects exhibited normal-range values and consistently lower measurements than both patient groups. Although liver biopsy was not performed in controls, their clinical and imaging profiles supported their inclusion as non-NASH subjects.

**Figure 3:**
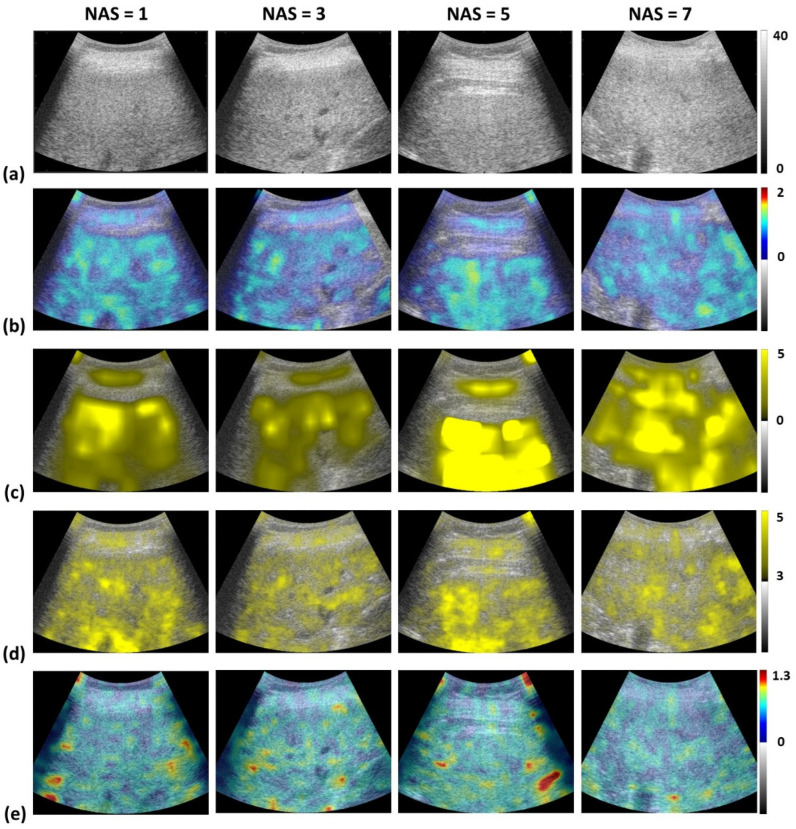
Representative (**a**) B-mode, (**b**) Nakagami, (**c**) HK, (**d**) entropy, and (**e**) attenuation images across different NAS levels. The QUS and attenuation images exhibited increased brightness with higher NAS, with visibly greater intensity in the NASH group compared to the non-NASH group. HK: homodyned-K; NAS: NAFLD activity score; QUS: quantitative ultrasound; NASH: nonalcoholic steatohepatitis.

**Figure 4:**
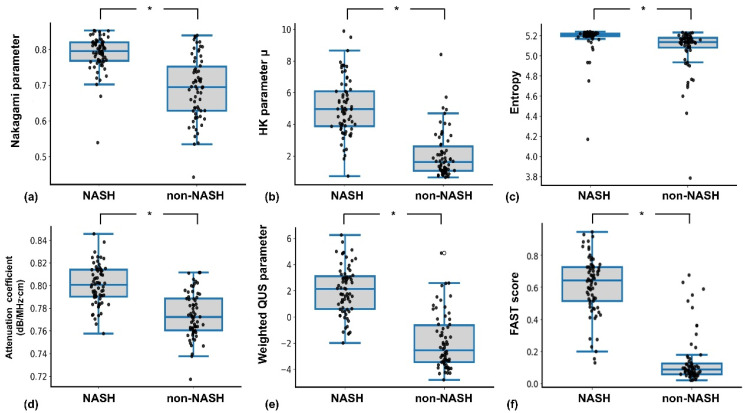
Box and scatter plots showing the distribution of each quantitative parameter in the non-NASH and NASH groups. (**a**) Nakagami parameter; (**b**) HK parameter; (**c**) entropy; (**d**) attenuation coefficient; (**e**) weighted QUS parameter; (**f**) FAST score. All parameters demonstrated statistically significant differences between groups (*: *p* < 0.05). HK: homodyned-K; QUS: quantitative ultrasound; NASH: nonalcoholic steatohepatitis; FAST: FibroScan–aspartate transaminase.

Figure 3 shows representative B-mode, envelope statistics, and attenuation images across NAS levels, with increasing image brightness correlating with higher NAS, especially in NASH cases. Figure 4 displays parameter distributions, all significantly elevated in the NASH group (*p* < 0.05). Compared to non-NASH subjects, the NASH group had higher Nakagami (0.79 ± 0.04 vs. 0.69 ± 0.087), HK (5.07 ± 1.81 vs. 2.06 ± 1.46), entropy (5.17 ± 0.14 vs. 5.06 ± 0.23), and attenuation coefficients (0.80 ± 0.01 vs. 0.77 ± 0.01 dB/MHz·cm). The weighted QUS parameter (2.04 ± 1.86 vs. −1.89 ± 2.10) and FAST score (0.61 ± 0.18 vs. 0.14 ± 0.15) were also significantly higher in NASH. Summary statistics are detailed in Table 2.

**Figure 5:**
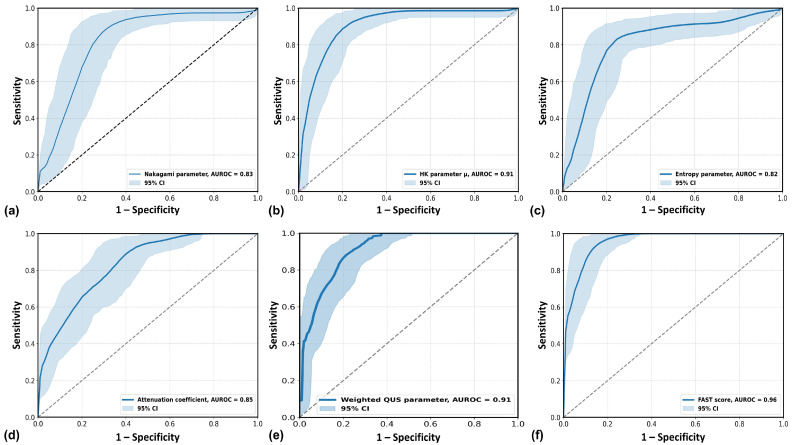
ROC curves with 95% confidence intervals (CIs) for each parameter in differentiating between non-NASH and NASH. (**a**) Nakagami parameter; (**b**) HK parameter; (**c**) entropy; (**d**) attenuation coefficient; (**e**) weighted QUS parameter; (**f**) FAST score. The AUROC values were 0.83 for Nakagami, 0.91 for HK, 0.82 for entropy, 0.85 for attenuation, 0.91 for the weighted QUS parameter, and 0.96 for the FAST score. ROC: receiver operating characteristic; HK: homodyned-K; QUS: quantitative ultrasound; NASH: nonalcoholic steatohepatitis; FAST: FibroScan–aspartate transaminase; AUROC: area under the ROC curve.

Figure 5 presents ROC curves for NASH classification. The AUROC values were highest for the FAST score (0.96), followed by HK and weighted QUS (both 0.91), with other QUS parameters ranging from 0.82 to 0.85. The optimal cutoffs were 0.75 (Nakagami), 3.41 (HK), 5.19 (entropy), 0.78 (attenuation), −0.13 (weighted QUS), and 0.41 (FAST). As shown in Table 3, HK and weighted QUS significantly outperformed other QUS features (*p* < 0.05) and showed no significant difference from the FAST score (*p* > 0.05, DeLong test).

Table 4 shows the separation metric results. The FAST score achieved the highest values across all four metrics, including 1–OVC (0.86), Bhattacharyya distance (0.97), KL divergence (11.63), and silhouette score (0.68). Among QUS parameters, the weighted QUS showed a strong performance (0.73, 0.49, 9.16, 0.50), comparable to HK (0.73, 0.43, 10.36, 0.48). Nakagami and attenuation showed moderate separation, while entropy performed the weakest.

## 4. Discussion

Recent studies have emphasized the importance of identifying high-risk NASH (fibrosis stage ≥ 2) to guide treatment decisions and prevent disease progression. The FAST score has shown promise as a noninvasive tool for risk stratification. Using cutoff values of 0.35 and 0.67, one study reported an AUROC of 0.80 for detecting high-risk NASH [8], while a meta-analysis confirmed pooled sensitivity and specificity of 89% at these thresholds [27]. Other studies have reported AUROCs ranging from 0.71 to 0.75 for identifying high-risk NASH or NAS ≥ 5 [28,29]. In one investigation, a FAST score ≥0.35 yielded a sensitivity of 96.4%, specificity of 36.8%, negative predictive value (NPV) of 77.7%, and positive predictive value (PPV) of 81.8% [30]. Collectively, these findings support the FAST score as a valuable noninvasive tool for identifying patients at risk of progressive NASH.

In our study, LSM values in the NASH group averaged (11.99 ± 10.40) kPa, suggesting that these subjects may be at a high-risk stage, as most studies indicate that LSM values above 7.8 kPa correspond to liver fibrosis ≥ 2 [31]. Under this condition, we evaluated the FAST score for NASH identification using our dataset. The FAST score cutoff we identified differed from those reported in previous studies, likely due to differences in cohort characteristics and sample size. Regardless of this slight variation, our findings align well with prior research, demonstrating that the FAST score provides outstanding performance in NASH detection, with an AUROC of 0.96. In comparison, QUS envelope statistics and attenuation imaging did not perform as well as the FAST score; however, their AUROC values remained clinically acceptable, suggesting they could serve as alternative approaches for NASH detection. These imaging techniques are particularly suitable for routine ultrasound examinations or community-based screening, where blood tests may not always be available. In such settings, ultrasound, with its high portability, could become a key tool for NASH detection, and the proposed quantitative imaging methods may offer a viable solution. Based on our findings, ultrasound HK imaging is recommended for ultrasound-based NASH detection, as its AUROC (0.91) was significantly higher than that of Nakagami (0.83), entropy (0.82), and attenuation (0.85) imaging.

The superior performance of the HK distribution in NASH detection may be attributed to its ability to establish a strong link between the properties of ultrasound backscattered echoes and the physical microstructure of tissue. Specifically, its *μ* parameter, estimated from envelope signals, is proportional not only to the number of scatterers within the transducer’s resolution cell but also to the homogeneity of scattering cross-sections [32] and the size of fat droplets [23]. Notably, the *μ* parameter has been shown to be particularly sensitive to hepatic steatosis, hepatocyte ballooning, and steatohepatitis [33], outperforming other envelope statistics parameters in characterizing pathological changes in the liver.

When compared to the HK parameter alone, the weighted QUS parameter achieved a comparable AUROC value (both 0.91), highlighting its similar discriminative capability for NASH detection. Although the HK parameter exhibited a slightly higher specificity (84.85% vs. 80.30%) and positive predictive value (PPV; 85.92% vs. 82.89%), the weighted QUS parameter demonstrated advantages in negative predictive value (NPV; 86.89% vs. 84.85%) and a more favorable negative likelihood ratio (LR−; 0.14 vs. 0.17). These findings suggest that the weighted QUS parameter may be more effective in reducing false-negative diagnoses, a critical consideration in clinical settings where undetected NASH poses a substantial risk. Also, based on statistical separation metrics, the weighted QUS parameter demonstrated improved separation between the non-NASH and NASH groups, as reflected by a higher Bhattacharyya distance (0.49 vs. 0.43) and silhouette score (0.50 vs. 0.48) compared to the HK parameter alone, further supporting the value of feature integration. The comparable performance of the weighted QUS parameter relative to the FAST score highlights its potential as a supplementary, or even standalone, feature in future classification models. Given its imaging-only basis, the weighted QUS approach offers a practical alternative to FibroScan and the FAST score, particularly in settings where blood-based testing is unavailable or where FibroScan performance is compromised (e.g., in cases of obesity or ascites). Its compatibility with standard ultrasound systems may also facilitate seamless integration into routine clinical workflows and community-based liver disease screening programs. In the future, QUS envelope statistics imaging and attenuation imaging may be integrated into clinical ultrasound systems, as both techniques primarily rely on beamformed backscattered signals and envelope data, which are already generated by conventional scanners. Provided that manufacturers allow access to these data, the methods could be implemented through software without requiring major hardware modifications.

This study has several limitations. First, the sample size is relatively small. Although we combined data from healthy volunteers and non-NASH patients into a single non-NASH group for comparison with the NASH group, potential bias in the analysis cannot be ruled out. Additionally, when determining diagnostic cutoff values for NASH based on various parameters and the FAST score, the cutoffs remained dependent on patient characteristics and sample size. Therefore, future large-scale clinical studies may be necessary to establish clinically applicable cutoffs with greater reliability. Moreover, this study was conducted at a single center, which may limit the generalizability of the findings due to restricted demographic and comorbidity diversity. Potential confounding effects of obesity, components of metabolic syndrome, and hepatic comorbidities on ultrasound backscatter and attenuation were also not fully accounted for. Future studies should include multicenter cohorts with more diverse populations and incorporate comprehensive adjustments for potential confounding factors to better validate the robustness and generalizability of the proposed methods.

In summary, this study supports the feasibility of extending QUS imaging from hepatic steatosis assessment to NASH detection. The weighted QUS parameter, integrating multiple acoustic features, showed strong discriminative performance second only to the FAST score. These results highlight the potential of multiparametric QUS as a noninvasive alternative for NASH detection in settings where FibroScan examination and blood-based tests (i.e., FAST) are limited, warranting further validation in larger, multicenter cohorts.

## Figures and Tables

**Table 1:** Demographic data of the participants enrolled in this study. The dataset, originally collected in a previous study [18], was reused under IRB approval for academic research purposes.

	Normal Participants	Non-NASH	NASH
No. of participants	49	17	71
Male/female	15/34	10/7	39/32
Demographics			
Age (years)			
Mean ± SD	56.65 ± 12.28 **	47.12 ± 11.57	50.28 ± 12.77 **
Median	59.00	49	54.00
Height (cm)			
Mean ± SD	160.40 ± 10.39	162.29 ± 7.83	164.01 ± 9.59 **
Median	159.00	163.00	163.00
Weight (kg)			
Mean ± SD	60.70 ± 12.22 **	71.36 ± 11.27	75.39 ± 13.90 **
Median	58.10	71.00	75.00
Anthropometrics			
BMI (kg/cm^2^)			
Mean ± SD	23.32 ± 2.79 **	27.11 ± 4.01	27.90 ± 3.65 **
Median	22.90	26.85	28.00
Liver function test			
AST (U/L)			
Mean ± SD	19.30 ± 8.26 *^,^ **	47.11 ± 31.74 *	72.32 ± 38.35
Median	18.00	39.00	60.00
ALT (U/L)			
Mean ± SD	25.04 ± 4.47 *^,^ **	72.65 ± 67.90 *	116.71 ± 73.02
Median	24.00	76.00	100.00
Histology			
Steatosis score			
Mean ± SD	N/A	1.12 ± 0.48	2.61 ± 0.56 **
Median		1.00	3.00
Hepatocyte ballooning score			
Mean ± SD	N/A	0.47 ± 0.51	1.46 ± 0.58 **
Median		0.00	2.00
Lobular inflammation score			
Mean ± SD	N/A	0.88 ± 0.49	1.67 ± 0.50 **
Median		1.00	2.00
FibroScan measurement			
LSM (kPa)			
Mean ± SD	3.78 ± 0.55 *^,^ **	6.38 ± 2.00	11.99 ± 10.40 **
Median	3.70	5.90	9.10
CAP (dB/m)			
Mean ± SD	210.57 ± 24.69 *^,^ **	287.82 ± 36.36	308.28 ± 45.28 **
Median	219.00	279.00	310.00

Note—NASH: nonalcoholic steatohepatitis; IRB: institutional review board; SD: standard deviation; BMI: body mass index; AST: aspartate aminotransferase; ALT: alanine aminotransferase; LSM: liver stiffness measurement; CAP: controlled attenuation parameter; N/A: not available; *: *p* < 0.05 compared to NASH; **: *p* < 0.05 compared to non-NASH.

**Table 2:** Comparison of QUS envelope statistics parameters (Nakagami, HK, and entropy values), attenuation coefficients, the weighted QUS parameter, and FAST scores between the non-NASH and NASH groups.

	Non-NASH	NASH
No. of participants	66	71
Male/female	25/41	39/32
Nakagami parameter		
Mean ± SD	0.69 ± 0.087	0.79 ± 0.04 *
Median	0.69	0.79
HK parameter *μ*		
Mean ± SD	2.06 ± 1.46	5.07 ± 1.81 *
Median	1.62	4.96
Entropy parameter		
Mean ± SD	5.06 ± 0.23	5.17 ± 0.14 *
Median	5.13	5.21
Attenuation coefficient		
Mean ± SD	0.77 ± 0.01	0.80 ± 0.01 *
Median	0.77	0.80
Weighted QUS parameter		
Mean ± SD	−1.89 ± 2.10	2.04 ± 1.86 *
Median	−2.54	2.14
FAST score		
Mean ± SD	0.14 ± 0.15	0.61 ± 0.18 *
Median	0.08	0.64

Note—NASH: nonalcoholic steatohepatitis; QUS: quantitative ultrasound; HK: homodyned-K; FAST: FibroScan–aspartate transaminase; SD: standard deviation; *: *p* < 0.05 compared to non-NASH.

**Table 3:** Performance evaluation of individual QUS parameters, the weighted QUS parameter, and the FAST score in NASH detection.

Parameter	Nakagami Parameter *m*	HK Parameter *μ*	Entropy Parameter *H*	Attenuation Coefficient *α*	LDA (*m*, *μ*, *H*, *α*)	FAST Score
Accuracy (%)	81.75	85.40	81.75	78.10	84.67	91.24
AUROC	0.83 *	0.91	0.82 *	0.85 *	0.91	0.96
Cutoff value	0.75	3.41	5.19	0.78	−0.13	0.41
Sensitivity (%)	88.73	85.92	83.10	87.32	88.73	91.55
Specificity (%)	74.24	84.85	80.30	68.18	80.30	90.91
LR+	3.44	5.67	4.22	2.74	4.50	10.07
LR−	0.15	0.17	0.21	0.19	0.14	0.09
NPV (%)	85.96	84.85	81.54	83.33	86.89	90.91
PPV (%)	78.75	85.92	81.94	74.70	82.89	91.55
*p* value (DeLong test) compared to FAST score	0.0008	0.105	0.0004	0.003	0.105	reference

Note—NASH: nonalcoholic steatohepatitis; QUS: quantitative ultrasound; HK: homodyned-K; FAST: FibroScan–aspartate transaminase; LDA: linear discriminate analysis; AUROC: area under the receiver operating characteristic curve; LR+: positive diagnostic likelihood ratio; LR−: negative diagnostic likelihood ratio; PPV: positive predictive value; NPV: negative predictive value. *: *p* value < 0.05 compared to HK parameter and the weighted QUS parameter.

**Table 4:** Results of four statistical separation metrics, including the complement of the overlap coefficient, Bhattacharyya distance, KL divergence, and silhouette score.

Parameter	Complement of Overlap Coefficient	Bhattacharyya Distance	KL Divergence	Silhouette Score
Nakagami parameter *m*	0.66	0.33	3.79	0.39
HK parameter *μ*	0.73	0.43	10.36	0.48
Entropy parameter *H*	0.60	0.09	1.16	0.10
Attenuation coefficient *α*	0.61	0.26	3.11	0.34
Weighted QUS parameter	0.73	0.49	9.16	0.50
FAST score	0.86	0.97	11.63	0.68

Note—HK: homodyned-K; FAST: FibroScan–aspartate transaminase; QUS: quantitative ultrasound; KL: Kullback–Leibler.

## Data Availability

The data presented in this study are available on request from the corresponding author.
